# Familial Melanoma and Susceptibility Genes: A Review of the Most Common Clinical and Dermoscopic Phenotypic Aspect, Associated Malignancies and Practical Tips for Management

**DOI:** 10.3390/jcm10163760

**Published:** 2021-08-23

**Authors:** Lamberto Zocchi, Alberto Lontano, Martina Merli, Emi Dika, Eduardo Nagore, Pietro Quaglino, Susana Puig, Simone Ribero

**Affiliations:** 1 Department of Medical Sciences, Dermatology Clinic, University of Turin, 10126 Turin, Italy; lamberto.zocchi@gmail.com (L.Z.); albertolontano@gmail.com (A.L.); merlimartina93@gmail.com (M.M.); pietro.quaglino@unito.it (P.Q.); 2 Dermatology, Department of Experimental, Diagnostic and Specialty Medicine, University of Bologna, 40138 Bologna, Italy; emi.dika3@unibo.it; 3 Department of Dermatology, Instituto Valenciano de Oncología, 46009 València, Spain; eduardo.nagoree@gmail.com; 4 Melanoma Unit, Dermatology Department, Hospital Clínic de Barcelona, IDIBAPS, Universitat de Barcelona, 08001 Barcelona, Spain; susipuig@gmail.com

**Keywords:** familial melanoma, *CDKN2A*, *MC1R*, *MITF*, *CDK4*, *POT1*, *BAP1*, dermoscopy, genetic, multiple melanoma

## Abstract

A family history of melanoma greatly increases the risk of developing cutaneous melanoma, a highly aggressive skin cancer whose incidence has been steadily increasing worldwide. Familial melanomas account for about 10% of all malignant melanomas and display an inheritance pattern consistent with the presence of pathogenic germline mutations, among which those involving *CDKN2A* are the best characterized. In recent years, a growing number of genes, such as *MC1R*, *MITF*, *CDK4*, *POT1*, *TERT*, *ACD*, *TERF2IP*, and *BAP1*, have been implicated in familial melanoma. The fact that individuals harboring these germline mutations along with their close blood relatives have a higher risk of developing multiple primary melanomas as well as other internal organ malignancies, especially pancreatic cancer, makes cascade genetic testing and surveillance of these families of the utmost importance. Unfortunately, due to a polygenic inheritance mechanism involving multiple low-risk alleles, genetic modifiers, and environmental factors, it is still very difficult to predict the presence of these mutations. It is, however, known that germline mutation carriers can sometimes develop specific clinical traits, such as high atypical nevus counts and specific dermoscopic features, which could theoretically help clinicians predict the presence of these mutations in prone families. In this review, we provide a comprehensive overview of the high- and intermediate-penetrance genes primarily linked to familial melanoma, highlighting their most frequently associated non-cutaneous malignancies and clinical/dermoscopic phenotypes.

## 1. Introduction

It is estimated that between 5% and 10% of malignant melanomas show familiarity [1]. The relative risk of melanoma, in fact, doubles in subjects with first-degree relatives with melanoma and increases with the number of affected family members. In addition, patients with familial melanoma often present with an early onset of melanomas and develop multiple primary melanomas [2]. Multiple primary melanomas tend to develop sporadically in 5% of those who had a melanoma, compared to 19% in patients who have a family history of melanoma [3]. Interestingly, a large number of these families also appear to have suffered from malignancies affecting other organs, falling under the so-called “multiple tumor syndrome” category [4].

Although some predisposing genes have been identified [5], not all cases of familial melanoma can be associated with pathogenic germline mutations, and the genetic basis of melanoma susceptibility remains unknown in a large number of families. Furthermore, the risk of developing melanoma in subjects with a predisposing mutation seems to correlate with the type of mutation, some environmental factors, and other unknown variables [6], suggesting a polygenic inheritance mechanism involving multiple low-risk alleles and genetic modifiers yet to be fully characterized [7]. One of such events is represented by epistasis, defined as the interaction between two genes where the effect of a particular gene depends on the presence of another modifying gene. Some epistatic mutations can, in fact, be found in the genesis of familial melanoma [8]. For example, some allelic variants of the melanocortin 1 receptor (*MC1R*) can act as genetic modifiers by increasing the penetrance of cyclin-dependent kinase inhibitor 2A (*CDKN2A*) mutations, doubling the risk of melanoma compared to those only harboring the *CDKN2A* mutation [9]. However, the hypothesis that heritable germinal epimutations may play a role in the formation of some familial clusters of melanoma, as shown for other neoplasms [10,11] is not supported by conclusive evidence [12].

The genes that predispose to melanoma are classified as low, medium, and high penetrance genes (Table 1) [13,14,15,16,17].

Each of these genes has a different intrinsic mechanism whose mutations can lead to the inactivation of fundamental biological mechanisms regulating cellular integrity. Alternatively, they can induce clinical phenotypic modifications exposing the subject to an increased risk of skin damage due to external factors such as photodamage in fair skin patients. Although these phenotypic modifications do not always predispose an individual to melanoma, they can potentially lead to the development of different traits, such as high nevus counts, larger congenital nevi and melanocytic lesions, characterized by well-defined clinical, dermoscopic, and histological features.

Among familial melanoma (with some exception) there is still lack of a clear correlation between certain mutations and a specific phenotype or dermoscopic feature. Establishing these correlations would be extremely important since it would enable oncologists to enroll predisposed subjects and their families into specific follow-up and screening programs for the timely detection of new tumors. In this regard, among the most potent predictors of a predisposing mutation are the number of melanomas developed by one subject and his/her family and the incidence of melanoma by geographical area. This aspect is particularly important given that genetic testing is highly recommended in those cases where the probability to find a predisposing mutation is higher or closer to 10%. Besides geographical prevalence and family history, the onset age of melanoma and its association with other neoplasms are also taken into account to determine the genetic predisposition to melanoma (Table 2) [18].

In this review, we provide a comprehensive overview of the genes most frequently involved in the development of familial melanoma, highlighting the clinical and dermoscopic phenotypes characterizing the patients with these mutations.

## 2. *CDKN2A*

### 2.1. Biology

*CDKN2A* was the first gene identified to be associated with a high risk of developing familial melanoma. It encodes two distinct proteins, p16 (INK4A) and p14 (ARF), both of which are tumor suppressors. The first arrests the cell cycle at the G1 phase via inhibition of *CDK4* and CDK6 kinases, while the latter enhances apoptosis and avoids oncogenic transformation [19]. Most *CDKN2A* mutations are p16-dependent and can be found in 10–25% of families with multiple melanomas in Australia, Europe and North America, which makes these mutations the most common cause of inherited susceptibility to melanoma [20].

The carriers of this mutation have an estimated risk of developing melanoma 87 times greater than the general population in UK and, for those living in Australia it is estimated to be 31 times greater [21]. The penetrance of *CDKN2A* mutations in these families is quite high, with an estimated 30% penetrance by age 50 years and 67% penetrance by age 80 years [22]. While the germline mutations of *CDKN2A* account for just 1–2% of melanoma cases in the general population [23], its prevalence increases with the number of primary melanomas diagnosed in a single patient and the presence of a familial history. In a multicenter Italian study [18], *CDKN2A* germline mutations were found in 19% of patients with multiple primary melanomas as opposed to 4.4% of patients with a single primary melanoma. The frequency of germline mutations also appears to correlate with the number of melanoma cases in a family [24]. For instance, in people with two or more relatives affected by melanoma, there is a 25% probability of detecting a germline *CDKN2A* mutation [23]. Furthermore, Goldstein et al. reported an overall mutational risk of 39% among families with three or more melanoma cases, ranging from 20% in Australia to 45% in North America and 57% in Europe [7]. In these families, the probability of harboring a germline *CDKN2A* mutation increases up to 74% in the presence of a diagnosis of pancreatic cancer [25].

The average age of diagnosis of melanoma in *CDKN2A*-mutated patients is between 30 and 40 years, whereas that of the general population is around 50 years. Although the onset of melanoma at a young age is quite common in subjects carrying *CDKN2A* mutations, it cannot be considered a predictor of the presence of the mutation; less than 1% of individuals diagnosed with melanoma before the age of 40 age are, in fact, positive for this mutation [6].

Although *CDKN2A* mutations confer an increased risk of melanoma, not all carriers of the mutation develop this pathology, suggesting that other environmental, clinical, and genetic factors contribute to increase the risk of melanoma [26,27,28]. Indeed, when assessing the relationship between geographical location and individual risk of bearing a *CDKN2A* mutation, it is important to take into account the variability of melanoma incidence in the general population and the degree of penetrance of *CDKN2A* mutations for each specific country considered [7,16,17]. It appears, in fact, that in regions with higher incidence of melanoma there is a greater possibility of finding multiple family members with melanoma or multiple primary melanomas caused by reasons other than *CDKN2A* mutations. As a matter of fact, a subject with more than four melanomas who is from a country with a low incidence of melanoma has between 29% and 100% chance of having this mutated gene. On the other hand, in a country with high incidence of melanoma such Australia, in families with even five affected members this probability does not exceed 10% [6]. Nevertheless, the fact that melanoma penetrance in *CDKN2A* mutation carriers is also higher in high-incidence areas indicates that there may be potential interactions between genetic factors and other predisposing factors in the carriers [6]. For example, the risk of developing melanoma in an 80-year-old individual with a family mutation of *CDKN2A* is increased by 58% in Europe, 76% in the US and 91% in Australia [22].

Another key element underscoring the multifactorial nature of melanoma is that, among families with *CDKN2A* mutations, family members harboring wild-type (wt) *CDKN2A* (i.e., phenocopies) tend to develop melanomas at a younger age compared to individuals with sporadic melanoma, even though the frequency of multiple primary melanomas in wt relatives still remains much lower compared to that of *CDKN2A* mutation carriers [29].

### 2.2. Associated Malignancies and Syndromes

In addition to melanomas, there exist many other tumors and syndromes associated with *CDKN2A* mutations, with pancreatic cancer being the most widely documented [30,31]. In particular, a Swedish study found that a particular *CDKN2A* mutation (p.Arg112dup) confers a 43.8 times higher risk of pancreatic cancer compared to the general population [26]. Fittingly, those with at least one melanoma and with either a personal or familial history of pancreatic cancer have a 9.7% chance of harboring a mutation in *CDKN2A* [32]. These latter are also at higher risk for malignancies of the upper digestive tract [26], respiratory tract [26,30] and breast [30,33]. Likewise, juvenile acute lymphoblastic leukemia (ALL) [34,35] and skin cancers, such as squamous cell carcinoma (SCC) and basal cell carcinoma (BCC) [26], are more likely to occur in *CDKN2A* mutation-bearing individuals. At 85 years of age, the risk of developing any of these non-melanocytic tumors is 59% [36]. In addition, this risk is significantly higher in smokers than in non-smokers [26,30].

The simultaneous loss of p14 and p16 gives rise to the so-called “melanoma-astrocytoma syndrome”, which is characterized by the presence of melanomas and astrocytomas and, occasionally, of other nervous-system neoplasms, such as peripheral nerve sheath tumors and meningiomas [37].

Germline mutations of *CDKN2A* have also been associated with familial atypical multiple mole melanoma syndrome (FAMMM), an autosomal dominant condition characterized by a family history of melanoma and a high number of atypical nevi [38].

It also appears that some single nucleotide polymorphisms (SNPs) in the 9p.21.3 region, which includes *CDKN2A*, may be linked to the development of type 2 diabetes mellitus, which appears to be a predisposing factor for pancreatic cancer [39].

### 2.3. Clinical Features

To date, it is not possible to predict the mutation status based on clinical observations. Some clinical features found in germline *CDKN2A* mutation carriers include a minor age at melanoma diagnosis, the presence of multiple primary melanomas [40], a tendency to develop superficial spreading melanomas in the absence of severe sunburns [41] and a very low proportion of acral and nodular melanomas [42]. Furthermore, there is no body-site concordance between *CDKN2A*-mutated familial melanomas [42], despite genetic has an impact on body-site distribution of naevi [43].

*CDKN2A*-mutation carriers have significantly more nevi than non-carriers and continue to develop additional nevi during their life. Moreover, existing nevi increase in size with time, and often develop one or more atypical clinical features. For this reason, these patients can have a clinical phenotype compatible with atypical nevus syndrome, defined by the presence of large numbers of nevi, and more than two atypical lesions [44]. Despite that, the great variability of phenotypic expression implies that not all mutation carriers display atypical nevi. Indeed, several studies report low mole counts and reduced number of atypical nevi in *CDKN2A* mutation carriers [45].

### 2.4. Dermoscopic Characteristics

From a dermoscopic viewpoint, unstructured areas are often observed in lesions of *CDKN2A*-mutated patients with two variants of *MC1R* of the RHC type, while streaks and pigmented networks are more frequently found in individuals without these variants [46]. In these subjects, incipient melanomas can be difficult to diagnose by dermoscopy alone as their poor color variegation and few dermoscopic structures resemble those of benign lesions [47]. Of note, in the nevi of *CDKN2A* G101W mutation carriers with at least one *MC1R* variant, the presence of vessels is more frequently associated with the absence of pigmentation [47,48].

### 2.5. Screening and Surveillance of Associated Malignancies

The screening and surveillance of high-risk *CDKN2A*-mutated patients remains a clinical research area in constant evolution. For patients with cutaneous melanoma, a surveillance program including skin, scalp, oral, and genital mucosa, an examination every three/six months (or annually based on the patient’s risk factors) is recommended. In this context, a dermoscopic comparative approach appears to be particularly useful, and sequential digital dermoscopic imaging can help diagnose early melanomas [49].

For those subjects suffering from pancreatic cancer and bearing a *CDKN2A* mutation, the guidelines issued by the American Cancer of the Pancreas Screening Consortium [50] and the American College of Gastroenterology [51] are still the most up-to-date resources. Initial screening should include an ultrasound (US) and/or magnetic resonance imaging performed annually starting from the age of 50 or 10 years earlier than the age of the earliest pancreatic cancer in the family [51].

For patients with melanoma-astrocytoma syndrome, it is highly recommended to perform regular brain imaging tests to monitor the development of astrocytoma alongside dermatological evaluation of the oral mucosa [37].

## 3. *MC1R*

### 3.1. Biology

The *MC1R* is a gene involved in human pigmentation, whose ligand is the α-melanocyte-stimulating hormone (α-MSH), a peptide derived from pro-opiomelanocortin (POMC). Binding of α-MSH to *MC1R* stimulates the preferential production of brown/black eumelanin compared to the red/yellow pigment of pheomelanin; the latter is potentially mutagenic due to its pro-oxidant effect [52,53,54]. The highest levels of pheomelanin are associated with the “red hair color” (RHC) phenotype (i.e., air skin, red hair, ephelides and sun light sensitivity) and it is caused by specific polymorphisms in the *MC1R* locus [55].

A study by Hernando et al. [56] described a different impact of the *MC1R* variants on the phototype of men vs. women, with the latter developing clearer phototypes compared to men carrying the same variant. Indeed, it seems that the increased production of female sex hormones can alter the effect of the RHC*-MC1R* variants on the skin phenotype and the risk of melanoma especially in young women [57].

Among individuals with darker phenotypes, the *MC1R* variants are associated with *BRAF V600E*-mutant melanomas on non-sun-exposed skin. Conversely, *MC1R* variants are inversely correlated with *BRAF V600K-*positive melanomas [58,59,60,61]. Furthermore, *MC1R* variants double the risk of melanoma in *CDKN2A* mutation carriers, which is three times higher in carriers of the RHC variants [62].

In addition to regulating skin pigmentation, *MC1R* signaling can also trigger DNA damage repair, thereby promoting cell survival. Thus, it is not surprising that polymorphisms of *MC1R* are associated with impaired DNA repair, leading to an increased susceptibility to the mutagenic effects of UV lights [63].

The observation that POMC-derived peptides, locally produced by keratinocytes and melanocytes, have immunomodulatory and anti-inflammatory properties has led to the hypothesis that the association between *MC1R* and non-melanoma skin cancers (NMSCs) may be the result of inflammatory or immune mechanisms influencing tumorigenesis [64,65], implying that not all *MC1R* mutations have to be necessarily associated with an RHC phenotype to be oncogenic (Table 3) [66].

Nonetheless, the risk of developing melanoma appears to be higher as the number of *MC1R* variants increases in the carrier; it doubles in subjects with a single variant and can go as high as six times in a subject with two or more variants [67,68,69,70].

The relationship between the presence of *MC1R* variants and the age of melanoma diagnosis is controversial. Whereas Box et al. [71] reported a significant reduction in the median diagnosis age in carriers of both *CDKN2A* mutations and *MC1R* variants compared to carriers of the *CDKN2A* mutation alone (37.8 years vs. 58.1 years), Chaudru et al. [72] and Van der Velden et al. [73] failed to detect any significant difference.

### 3.2. Associated Malignancies and Syndromes

Individuals carrying any *MC1R* variant have a significantly higher risk of developing NMSCs compared to subjects without these variants [53]. Specifically, the presence of at least one *MC1R* variant caused a higher risk of SCC (OR = 1.61) and BCC (OR = 1.39). Subjects with two or more variants present an increased risk of developing BCC (OR = 1.70) and SCC (OR = 2.10) compared to carriers of one *MC1R* variant. A significant *MC1R*-associated NMSC risk was observed only among subjects without the RHC phenotype, suggesting that *MC1R* can contribute to skin tumorigenesis through different mechanism other than pigmentation [53]. Finally, it seems that there is an independent association between *MC1R*, hair, and skin color, nevi, UV photodamage and the risk of developing this type of tumor.

### 3.3. Clinical Features

The RHC variants are associated with fair skin and red hair and, depending on the number of variants present in each individual, with the presence of ephelides and solar freckles [74].

No association was found between the *MC1R* variants and high counts of acquired nevi in families with hereditary melanoma [70]. Two studies have documented a lower number of acquired melanocytic nevi in juvenile subjects with red hair, especially those with a large number of ephelides [75,76]. Furthermore, despite most studies have failed to show an effect of *MC1R* on the eye color, the R142H variant seems to correlate with a blue/green eye color [77], implying a potential epistatic interaction between the eye color-determining genes *MC1R* and *OCA2* [75,77,78].

In good agreement with Palmer et al. [67], a meta-analysis by Kanetsky et al. [79] unveiled that the risk of melanoma associated with *MC1R* variants is higher in individuals with protective phenotypes and limited sun exposure compared to patients with fair skin. This seems to confirm that *MC1R* gene may play a role in the pathogenesis of melanoma not only through the synthesis of melanin associated with the sun exposure, but also through a direct effect on melanocyte cellular transformation.

Concerning the body site location, patients with RHC-*MC1R* variants tend to develop melanomas mainly on the trunk and the arms [80,81]. Furthermore, the carriers of this variants generally develop hypopigmented melanocytic lesions, called “white nevi” or “red melanomas” [48] and display larger nevi and melanomas compared to those of wt individuals [82,83].

It has been also observed that some genotypes of *MC1R* can increase the number of nevi with a diameter larger than >2 mm as well as of larger congenital melanocytic nevi. However, a consequent increase in total nevus count has not been detected, indicating that the formation of nevi with different sizes occurs through multiple mechanisms [84,85]. Noteworthy, this data has not been confirmed in a recent multicenter study [86].

### 3.4. Dermoscopic Characteristics

*MC1R* variants, especially the RHC one, are known to modulate the color and the dermoscopic pattern of melanomas and nevi, resulting in hypopigmentation and the presence of fewer dermoscopic structures, which highlight the vascular pattern of these tumors [46,47,48]. Indeed, nevi from individuals with no RHC variants contained significantly more streaks, pigment networks and number of colors, while nevi from individuals with 2 RHC variants contained a larger number of structureless areas with lighter colors [46]. By contrast, Vallone et al. [82] observed the presence of nevi with an eccentric hyperpigmentation and the formation of globules.

In two different studies, melanomas from RHC-*MC1R* carriers showed lower values of the ABCD Total Dermoscopy Score (TDS) compared to the carriers without the RHC variant. This observation might be explained by the fact that melanomas carriers of *MC1R* variants have a relatively low number of colors and structures, which makes these individuals unsuitable for TDS scoring [47,87]. In good agreement, Longo et al. [88] showed that early melanomas in *CDKN2A* mutation carriers with RHC variants are more difficult to diagnose, even when employing a comparative approach aimed to evaluate the single lesion in the context of the patient’s overall nevus profile. Therefore, they seem to benefit from changes detection during digital dermoscopy monitoring for early diagnosis.

### 3.5. Screening and Surveillance of Associated Malignancies

Currently, there is no evidence available.

## 4. *MITF*

### 4.1. Biology

The melanocyte inducing transcription factor (*MITF*) gene encodes for a transcription factor involved in cell pigmentation and development and differentiation of melanocytes, limiting their proliferation through the expression of p16, p21, and other anti-apoptotic genes such as *BCL2* and *APEX1* [89].

The most common variant of *MITF* is *p.E318K*, which alters its binding affinity for the ubiquitin-like protein SUMO, thus enhancing the binding of *MITF* protein to the HIF1A promoter and increasing its transcriptional activity [90,91]. The studies collected so far have shown *MITF E318K* germline mutations to be associated with a 2.2 to 5 times higher risk of developing melanoma compared to the general population [90,91,92].

### 4.2. Associated Malignancies and Syndromes

In addition to melanoma, germline mutations of *MITF* have been associated with other malignancies. For instance, the germline mutation *E318K* confers a more than five-fold increased risk of developing melanoma, renal cell carcinoma or both cancers [90]. Carriers with either a personal or familial history of pancreatic or kidney cancer have a nearly 31- and 8-fold higher risk of developing skin melanomas compared to wt individuals [92]. Finally, the *p.E318K* variant of the *MITF* gene is related to a higher risk of developing pheochromocytomas and paragangliomas compared to the general population [93].

### 4.3. Clinical Features

The analysis of the phenotypic traits of patients with *MITF* germinal mutations has revealed an association of this mutation with high mole count, onset of skin melanoma before the age of 40 and non-blue eye color. By contrast, no association was found with the presence of freckles, phototype or hair color [91]. These observations were confirmed by another study [94] reporting a higher incidence of amelanotic melanomas in patients with fair skin, who not only displayed a high number of nevi but also had more nevi with a diameter greater than 5 mm compared to controls. The color of these nevi was pink or light brown, suggesting a role of the *MITF* mutation in modulating the pigmentation of the nevi [94]. Ciccarese et al. showed that germline mutations of *MITF* are associated with a higher frequency of development of nodular melanomas [95], which were mainly localized on the back, followed by leg, arm and abdomen, all areas that can be intermittently exposed to UV rays [94]. The role of a potential interaction between the RHC*-MC1R* variants and the *MITF* variant in the pigmentation of melanoma (amelanotic or pigmented) was evaluated, but no association was identified [96].

### 4.4. Dermoscopic Characteristics

Some studies have found that the predominant dermoscopic pattern of the nevi in patients carrying the *MITF p.E318K* mutation is the reticular pattern [15,94,97]. Recently, Ciccarese et al. [95] has described three prevalent dermoscopic patterns in dysplastic nevi and melanomas: (i) non-specific; (ii) globular-homogeneous; and (iii) reticular-homogeneous. As for melanomas alone, the prevalent pattern in *MITF*-mutated patients is the nonspecific one, including amelanotic/hypomelanotic nodular melanomas, where it can be associated with atypical polymorphic vessels.

### 4.5. Screening and Surveillance of Associated Malignancies

There is currently no evidence available.

## 5. *CDK4*

### 5.1. Biology

The cyclin dependent kinase 4 (*CDK4*) is an oncogene that mediates progression through the G1 phase, when cells are preparing for the DNA synthesis step. All *CDK4* mutations occur on codon 24, with *CDK4*-mutated families carrying the Arg24His (R24H) or Arg24Cys (R24C) substitution. As the arginine in this position is required for p16 binding to *CDK4*, its substitution with histidine or cysteine hampers the ability of p16 to inactivate *CDK4*-mediated G1/S transition [98].

Germline mutations of *CDK4*, albeit quite rare, predispose carriers to melanoma development to a similar extent to that observed in individuals harboring *CDKN2A* mutations [98]. More specifically, 41.7% of melanoma patients from 17 families carrying *CDK4* mutations (R24H in 11 families and R24C in 6 of them) developed more than one primary tumor [98], with the tumor number ranging from 2 to 13. The mean age of diagnosis of the first melanoma was 39 years, which is about 15 years earlier than that of the general Caucasian population. In addition, only 7.4% received diagnoses at the age of 60 or above with an estimated lifetime mutation penetrance of 74.2% at the age of 50 years [98].

### 5.2. Associated Malignancies and Syndromes

Families carrying the germline mutation of *CDK4* had a higher risk of developing breast cancer and NMSCs [98], even though the onset of these tumors was reportedly like that seen among sporadic cases.

### 5.3. Clinical Features

Puntervoll et al. reported a greater onset of melanoma in the fourth decade of life and similar median age of first diagnosis among males and females harboring the *CDK4* mutations [98]. In these patients, the predominant histologic type was superficial spreading melanoma (SSM), and most of the tumors were located on the limbs. Finally, there was no difference in the color of the skin and hair between mutated and non-mutated patients, while the number of atypical nevi was higher in mutated patients [98].

### 5.4. Dermoscopic Characteristics

To date, there is no evidence available.

### 5.5. Screening and Surveillance of Associated Malignancies

To date, there is no evidence available.

## 6. *POT1*

### 6.1. Biology

The protection of telomeres 1 (*POT1*) gene encodes for a shelterin complex protein, which plays an essential role in preserving telomere integrity and DNA replication. The shelterin complex consists of six family members, encoded by the following genes: *POT1*, *ACD*/*TPP1*, *TERF1/TRF1*, *TERF2/TRF2*, *TERF2IP/RAP1,* and *TINF2/TIN2* [99].

*POT1* binds single-stranded telomeric DNA thanks to two N-terminal oligonucleotide-/oligosaccharide-binding (OB) domains, through which it mediates access to the telomerase complex [100]. *POT1* loss-of-function germline mutations associated with melanoma arise in the OB region of the gene. These mutations cause insufficient capping of the telomeres by the shelterin complex, abnormal telomeric length and possible chromosomal fusions, which hide the telomeric signal [101,102].

Robles-Espinoza et al. reported that all nine *POT1* variant carriers from the familial cohort analyzed had developed at least one melanoma (for a maximum of 8) from 25 to 80 years of age [101]. Histological studies of some melanomas in *POT1*-mutated families showed a predominance of the spitzoid morphology, suggesting that telomere dysfunction may contribute to this type of differentiation [103].

### 6.2. Associated Malignancies and Syndromes

Some germline variants of *POT1* have also been described in families with chronic lymphatic leukemia [104], cardiac angiosarcoma and other tumor types in TP53-negative Li-Fraumeni-like syndrome [105], colorectal cancer [106], thyroid neoplasms [107], gliomas [108], breast cancer, and endometrial and small cell lung carcinomas [101]. However, since none of these variants have been described in melanoma patients, it is likely that each corresponding mutation is tumor specific.

### 6.3. Clinical Features

There is currently no evidence available.

### 6.4. Dermoscopic Characteristics

There is currently no evidence available.

### 6.5. Screening and Surveillance of Associated Malignancies

Considering the increased risk of thyroid neoplasms in *POT1* germline mutation carriers, Wilson et al. [107] recommend these patients be subjected to thyroid US follow-up every one to two years in addition to regular six-month dermatological checks and age-appropriate tumor screening. Given the early onset of melanomas in mutated patients, it is also recommended that these genetic tests should be continuously performed throughout childhood.

## 7. *TERT*

### 7.1. Biology

The telomerase reverse transcriptase gene *(TERT*) encodes another protein belonging to the shelterin complex. It is a reverse transcriptase that, together with the product of the telomerase RNA component (*TERC*) gene, constitutes the main component of telomerase [109].

Mutations in the *TERT* promoter region have been described at both the somatic and germline level [109], and they seem to provoke excessive elongation of telomeres, a factor that could lead to telomerase activation and, therefore, to increased cell survival, allowing an accumulation of oncogenic somatic mutations over time [110]. Germline mutations in *TERT* are rare in familial melanomas and are responsible for approximately 1% of familial melanomas [111].

### 7.2. Associated Malignancies and Syndromes

*TERT* has been reported to be somatically mutated in 1% of cancers including melanoma, where it represents a UV-signature [112]. Furthermore, the germline mutation of *TERT* is associated with an increased risk of developing chronic myeloproliferative neoplasms [113,114]. *TERT*-mutated patients have also an increased probability of developing cancer in the bladder [115], colon [116], lung [117], prostate [118], testis [119], breast [120], kidney [121], and central nervous system (i.e., glioblastoma, glioma, and medulloblastoma) [117].

Germinal mutations can predispose to many familiar liver diseases, such as steatosis [122], cirrhosis and hepatocellular carcinoma [123]. In a percentage ranging from 8 to 15% of familial cases, it can also predispose to idiopathic pulmonary fibrosis (IPF), with an increased risk of lung cancer [124,125,126]. However, the association of these pathologies with melanoma remains unclear.

### 7.3. Clinical Features

Few data are available on the clinical phenotype of patients with germline mutations of the *TERT* gene, although they tend to display high nevi counts [127].

### 7.4. Dermoscopic Characteristics

Regarding dermoscopy, *TERT* germline mutations have been associated with globular mole pattern [128].

### 7.5. Screening and Surveillance of Associated Malignancies

There is currently no evidence available.

## 8. *ACD* and *TERF2IP*

### 8.1. Biology

In addition to *POT1* and *TERT* mutations, other mutations altering the function of the shelterin complex have been described, including those of the adrenocortical dysplasia (*ACD*) and the telomeric repeat-binding factor 2-interacting protein 1 (*TERF2IP*) genes.

ACD not only increases the affinity of *POT1* for single-strand telomeric DNA but, together with *POT1*, also mediates the interaction of the shelterin complex with *TERT* [99].

On the other hand, *TERF2IP* is necessary to determine a further repression of homologous repair of double strand chromosome breaks at the telomeres level. Additionally, *TERF2IP* can associate with the NF-κB kinase inhibitor complex (IKK), by regulating the NF-κB signaling pathway. Finally, it can bind other extra-telomeric chromosomal regions and interact with other factors, participating in the silencing of sub-telomeric genes responsible for metabolism and genomic stability [129,130].

The main germline *ACD* and *TERF2IP* mutations are nonsense mutations associated with increased risk of multiple primary melanomas and earlier age of melanoma diagnosis, with a peak in the second decade [99].

### 8.2. Associated Malignancies and Syndromes

Other malignancies have been identified as possible carriers of *ACD* and *TERF2IP* germline mutations, including lung, breast [99], colorectal [131] and hematological malignancies (B cell lymphoma and leukemia) [99], as well as progression to bone marrow failure with possible aplastic anemia [132].

### 8.3. Clinical Features

There is currently no evidence available.

### 8.4. Dermoscopic Characteristics

There is currently no evidence available.

### 8.5. Screening and Surveillance of Associated Malignancies

There is currently no evidence available.

## 9. *BAP1*

### 9.1. Biology

*BAP1* is a gene encoding the BRCA1 associated protein 1 (BAP1) that plays a central role in numerous cellular processes, including cell division, gene expression, signal transduction, protein trafficking, DNA repair and regulation of DNA repair, all pathways whose dysregulation can promote oncogenesis. For example, *BAP1*, which can act as a UV ray-inducible substrate of the serine/threonine kinases ataxia telangiectasia-mutated (ATM) and Rad3-related (ATR), involved in cell growth arrest, DNA repair and apoptosis, has been shown to regulate melanocytic differentiation [133].

Uveal melanoma is associated with *BAP1* mutations in almost 3% of the cases [134,135]. Njauw et al. [134] reported that 29% of families with combined cases of cutaneous melanomas and uveal melanomas had mutations in *BAP1* as opposed to families with multiple cutaneous melanomas alone where *BAP1* mutations were present in just 0.52% of cases. Lastly, *BAP1* mutations occur with greater frequency among metastatic ocular melanoma (OM) patients compared to their non-metastatic OM counterparts [134].

### 9.2. Associated Malignancies and Syndromes

*BAP1* germline mutations predispose carriers to a high number of tumors, with a high degree of intrafamily variability, which may indicate an interplay with modifying genes or environmental factors.

The first tumor syndrome described in *BAP1* family mutations was the complex of cutaneous/ocular melanoma, atypical melanocytic proliferation, and other internal neoplasms (COMMON syndrome) [133,134]. This complex of tumors is phenotypically characterized by the appearance of clinically benign, albeit histologically atypical, melanocytic skin tumors at a young age, and by the high incidence of mesothelioma (their onset occurs 20 years earlier than the average age even in the presence of low asbestos exposure [136]), uveal melanoma, cutaneous melanoma, and other neoplasms [133].

Other tumors associated with *BAP1* germline mutations include renal cell carcinoma [137,138], lung carcinoma [139], meningioma [140], paraganglioma [141], cholangiocarcinoma [142], breast tumor, neuroendocrine carcinoma, gastric tumors [141], papillary adenoma and thyroid carcinomas [143], BCC [144] and SCC [145]. It is estimated that 63.5% of patients with *BAP1* germline mutations develop at least one of these tumors at an age younger than that of the general population (53.2 years) [146].

In cases where several family members suffer from multiple metastatic carcinomas with unknown primary tumors, it should be considered that the family they belong to may carry *BAP1* germline mutations [147]. This aspect is of particular relevance given that *BAP1* mutations are generally associated with a worse prognosis for patients with cutaneous melanoma, uveal melanoma and renal cell carcinoma [148]. In contrast, the presence of mesothelioma is associated with a survival up to seven times higher than that seen in sporadic cases of melanomas [136]. Uveal melanomas are larger in size and involve predominantly the ciliary bodies, two clinical factors correlated with poor prognosis due to their higher metastatic potential [149]. *BAP1* mutations are also regarded as a risk factor for leptomeningeal melanocytic tumors [150].

### 9.3. Clinical Features

The average age of diagnosis of uveal melanoma in the carriers of this mutation is lower than that of the general population (53 vs. 62 years) [151,152], with an earlier onset even before the age of 20 [141,153,154,155].

These patients tend to develop a greater number of atypical melanocytic lesions [156], known as atypical Spitz tumors [157] or nevoid melanoma-like melanocytic proliferations (NEMMPs) [134], which are all usually referred to as multiple *BAP1*-inactivated melanocytic tumors (BIMTs). Histology shows nevus-like lesions usually formed by a conventional junctional, compound, or dermal nevus composed of small melanocytes expressing *BAP1*. Next to these, there is a dermal lesion formed by large epithelioid *BAP1* negative and most frequently BRAF mutated melanocytes with virtually no mitotic activity [151].

These are basically a heterogeneous group of nodular melanocytic tumors, dome-shaped, skin-colored to reddish-brown, resembling intradermal nevi or fibromas, with an average size ranging from 5 mm to 6.85 mm. They can rarely appear as brown papules/macules [157,158] or tan macules [159]. In addition, they appear in variable numbers (from 5 to over 50) [146,160] and are frequently located on the head and neck, followed by the trunk and the limbs [159].

BIMTs appear from the second decade of life—the average age at diagnosis is of 36.9 years [159]. For this reason, together with the appearance of other gene-related tumors, the multiple presence of these lesions could be used as markers for the presence of *BAP1* germline mutations.

### 9.4. Dermoscopic Features

Thanks to anecdotal reports, all BIMTs can be divided in five dermoscopic patterns: (1) structureless pink-to-tan with irregular dots/globules located eccentrically [161]; (2) structureless pink-to-tan with peripheral vessels [162,163,164]; (3) structureless pink-to-tan; (4) network with raised, structureless, pink-to-tan areas; and (5) globular pattern [159]. The first and the fourth pattern seem to be more frequent in patients with multiple BIMTs associated with a known *BAP1* germline mutation. On the other hand, the globular pattern seems to be a negative predictor of *BAP1* germline mutations [159].

### 9.5. Screening and Surveillance of Associated Malignancies

According to a comprehensive review by Rai et al. [165], it is very important for *BAP1*-mutated patients to perform periodically the following evaluations:

1. An annual ophthalmological evaluation from 16 to 30 years of age and then every 6 months after the age of 30. An indirect ophthalmoscopy, a fundus photograph and an ocular US should also be performed during the visit;
2. A dermatological evaluation with a total body skin examination every 6 months starting from the age of 18;
3. Annual examination of the abdomen and chest from the age of 30, looking for abdominal masses, abdominal distention, ascites or signs of pleural effusion. Between the ages of 30 and 55, it should be offered the opportunity to perform US or MRI scans of the thoraco-abdominal and urinary tract areas every 2 years. After the age of 55, a thoraco-abdominal CT or MRI scan, both with contrast media, should be performed every 2 years. A renal and chest US may be considered in the interval between CT/MRI.

## 10. Conclusions

To date, it is known that familial melanoma can be associated with an inheritance pattern of high/moderate-penetrance germline mutations in predisposing genes. Despite *CDKN2A* variants are the most known, recently other genes variants, such as *MC1R*, *MITF*, *CDK4*, *POT1*, *TERT*, *ACD*, *TERF2IP*, and *BAP1,* have turned out to play an important role in genetic susceptibility to cutaneous melanoma. Moreover, these genes are fundamental in several biological mechanisms, so it is not surprising that their variants have been associated with a broader range of other malignancies. Understanding association between gene mutations and cancer risk is of great help for clinicians involved in cancer screening: it can guide the clinician in performing appropriate genetic testing and surveillance in these families.

Dermatologists should be aware that germline mutation carriers can sometimes develop specific phenotypes, also including specific dermoscopic features, which could support in identifying melanoma-prone families. Nevertheless, we need further studies that better characterize this genotype-phenotype correlation leading to optimal management of these patients.

## Figures and Tables

**Table 1:** Main genes associated with the development of familial melanoma, relative prevalence and function.

	Genes	Prevalence in Familial Melanoma	Functions of Coded Proteins
High penetrance genes	*CDKN2A*	20–40%	Encodes 2 tumor-suppressor proteins
	*CDK4*	<1%	Cell cycle regulator
	*POT1*	<1%	Telomere maintenance
	*ACD*	<1%	Telomere maintenance
	*TERF2IP*	<1%	Telomere maintenance
	*TERT*	<1%	Telomere maintenance
	*BAP1*	<1%	Tumor suppressor
Intermediate penetrance genes	*MC1R*	70–90%	Melanin production
	*MITF*	1–5%	Regulation of melanocyte development

**Table 2:** Guidelines for carrying out the genetic test according to the incidence of melanoma in different countries. The development of multiple primary melanomas, regardless of regional differences, can be considered a single criterion for access to genetic testing, even in the absence of a family history of melanoma.

Incidence of Melanoma by Geographical Area	Criteria for Access to Genetic Testing
Low moderate incidence areas (e.g., Italy) 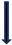 RULE OF 2	Two primary melanomas (synchronous or metachronous) in an individual and/or Families with at least one invasive melanoma and 1 or more other diagnoses of melanoma and/or pancreatic cancer among first or second-degree relatives on the same side of the family
Moderate/high incidence areas (e.g., US) 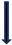 RULE OF 3	Three primary melanomas (synchronous or metachronous) in an individual and/or Families with at least one invasive melanoma and 2 or more other diagnoses of melanoma and/or pancreatic cancer among first or second-degree relatives on the same side of the family
Very high incidence areas (e.g., Australia) 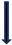 RULE OF 4	Four primary melanomas (synchronous or metachronous) in an individual and/or Families with at least one invasive melanoma and 3 or more other diagnoses of melanoma and/or pancreatic cancer among first or second-degree relatives on the same side of the family

**Table 3:** Correlation between *MC1R* variants and melanoma risk, RHC phenotype and increased risk for BCC and SCC.

Variants	Melanoma Risk	Association with RHC Phenotype	Increased Risk for BCC	Increased Risk for SCC
R151C	+	+	+	+
R160W	+	+	+	+
D294H	+	+	+	+
D84E	+	+	+	−
R142H	+	+	−	−
V60L	−	−	+	+
V92M	−	−	+	+
R163Q	+	−	−	−
I155T	+	−	−	−

+: presence of correlation; −: absence of correlation.
